# Recent Advances in BLV Research

**DOI:** 10.3390/v7112929

**Published:** 2015-11-24

**Authors:** Pierre-Yves Barez, Alix de Brogniez, Alexandre Carpentier, Hélène Gazon, Nicolas Gillet, Gerónimo Gutiérrez, Malik Hamaidia, Jean-Rock Jacques, Srikanth Perike, Sathya Neelature Sriramareddy, Nathalie Renotte, Bernard Staumont, Michal Reichert, Karina Trono, Luc Willems

**Affiliations:** 1 Molecular and Cellular Epigenetics (GIGA) and Molecular Biology (Gembloux Agro-Bio Tech), University of Liège (ULg), Liège 4000, Belgium; epy.barez@doct.ulg.ac.be (P.-Y.B.); alix.debrogniez@ulg.ac.be (A.B.); a.carpentier@doct.ulg.ac.be (A.C.); helene.gazon@ulg.ac.be (H.G.); n.gillet@ulg.ac.be (N.G.); mhamaidia@ulg.ac.be (M.H.); jacques.jeanrock@gmail.com (J.-R. J); Srikanthperike@gmail.com (S.P.); sathy.ns@gmail.com (S.N.S.); nrenotte@ulg.ac.be (N.R.); b.staumont@doct.ulg.ac.be (B.S.); 2 Instituto de Virología, Centro de Investigaciones en Ciencias Veterinarias y Agronómicas, INTA, Castelar C.C. 1712, Argentina; gutierrez.geronimo@inta.gob.ar (G.G.); trono.karina@inta.gob.ar (K.T.); 3 Department of Pathology, National Veterinary Research Institute, Pulawy 24-110, Poland; reichert@piwet.pulawy.pl

**Keywords:** BLV, HTLV-1, Tax, microRNA, vaccine, HDAC

## Abstract

Different animal models have been proposed to investigate the mechanisms of Human T-lymphotropic Virus (HTLV)-induced pathogenesis: rats, transgenic and NOD-SCID/γcnull (NOG) mice, rabbits, squirrel monkeys, baboons and macaques. These systems indeed provide useful information but have intrinsic limitations such as lack of disease relevance, species specificity or inadequate immune response. Another strategy based on a comparative virology approach is to characterize a related pathogen and to speculate on possible shared mechanisms. In this perspective, bovine leukemia virus (BLV), another member of the deltaretrovirus genus, is evolutionary related to HTLV-1. BLV induces lymphoproliferative disorders in ruminants providing useful information on the mechanisms of viral persistence, genetic determinants of pathogenesis and potential novel therapies.

## 1. Introduction

BLV naturally infects cattle, zebu and water buffalo but can also be experimentally transmitted to sheep, goats or alpaca (Vicugna pacos) [1,2,3]. In cattle, the most prevalent clinical manifestation is a benign accumulation of infected B-lymphocytes called persistent lymphocytosis (PL) affecting about one-third of infected animals [4,5]. In a minority of cases (about 5%–10%), BLV infection can progress to fatal leukemia/lympoma whose most spectacular consequence is spleen disruption consecutive to tumor formation [6]. BLV typically persists in less than 1% of peripheral blood cells, leading to an asymptomatic infection in the majority of infected animals. BLV is transmitted horizontally by direct contact, iatrogenic procedures or insect bites upon transfer of infected cells from milk, blood and body fluids from heavily infected dams [7,8].

Among experimental hosts, sheep provide a useful model to address specific questions pertaining to immunity, viral persistence and pathogenesis. In particular, reverse genetics permitted the development of a life-attenuated vaccine and a novel therapeutic approach. Main advantages of the sheep model include a high frequency of leukemia/lymphoma (close to 100%) and a shorter latency period (typically 2–4 years).

BLV-associated pathogenesis thus shares a series of features with HTLV-1-induced Adult T-cell Leukemia (ATLL) but does apparently not include neurodegenerative diseases such as HTLV-Associated Myelopathy/Tropical Spastic Paraparesis (HAM/TSP) [9]. It is assumed that consumption of raw milk from BLV-infected cattle is not associated with an increased risk of cancer in human, although the link cannot be formally excluded [10].

The goal of this review is to outline interesting observations in the BLV model that are of interest to understand HTLV-1 replication and pathogenesis.

## 2. Viral Oncogenes Drive Proliferation

**Figure 1:**
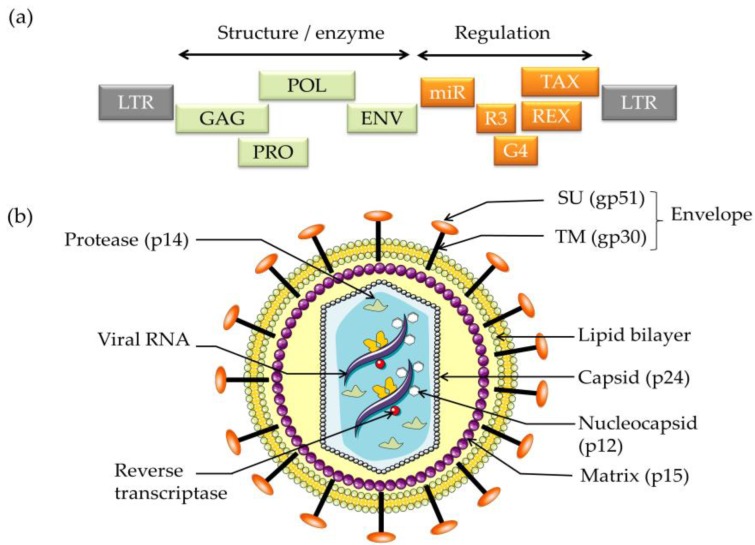
Schematic structure of (**a**) the bovine leukemia virus (BLV) genome and (**b**) the viral particle.

As deltaretrovirus, BLV carries the classical genes (*gag*, *pro pol* and *env*) that are required to complete the viral cycle: genesis and budding of a virion, infection of a target cell, reverse transcription and integration into the host cell chromosome. The BLV provirus also encodes a series of additional accessory genes as well as microRNAs that modulate viral and/or cellular gene expression (Figure 1) [11,12]. Among these, Tax and G4 are oncogenes able to promote transformation of primary rat embryo fibroblasts [13,14]. Tax activates transcription by acting on a triplicate 21 bp enhancer motif in the 5′ the LTR promoter via the CREB/ATF signaling pathway [15,16]. Although, the mechanisms of cell transformation remain to be further characterized, it is interesting to note that BLV and HTLV-1 Tax share cellular targets. Both transactivators indeed bind to tristetraprolin (TTP), a post-transcriptional modulator of TNFα expression [17]. The Tax proteins promote nuclear accumulation of TTP and restore TNFα expression by inhibiting TTP.

Another cellular protein concomitantly targeted by BLV G4 and its ortholog in HTLV-1 (p13) is farnesyl pyrophosphate synthase (FPPS), an enzyme involved in the mevalonate/squalene pathway and in synthesis of FPP, a substrate required for prenylation of Ras [18]. In addition FPPS is involved in synthesis of isoprenoids modulating the membrane fluidity and stability of lipid rafts [19]. Being localized in the nuclear compartment and in mitochondria, G4 and p13 thus exert evolutionary conserved functions.

Recently, a proviral region located 3′ of the env gene was shown to express microRNAs under the control of RNA polymerase III promoters (miR on Figure 1) [20]. The BLV microRNAs associate with Argonaute and mimic cellular analogs (e.g., BLV-miR-B4 for miR-29). BLV microRNAs are transcribed from a region dispensable for *in vivo* infectivity but are abundantly expressed in leukemic B cells (about 40%) [21]. This evidence thus contradicts the dogma that naturally occurring RNA viruses will not encode miRNAs to avoid unproductive cleavage of their genomes. HTLV-1 does not encode microRNAs as indicated by deep sequencing. The role of the BLV microRNAs in viral replication, persistence and disease remains to be further characterized.

BLV thus encodes transformation drivers (Tax, G4) and viral microRNAs likely important in pathogenesis.

## 3. Reverse Genetics Reveals the Significance of Viral Sequences in Infection and Replication

Reverse genetics using a cloned BLV provirus has allowed the screening of regions required for infection, replication and pathogenesis. As expected, large deletions within the *gag*, *pol* or *env* genes destroy infectivity *in vivo*. Discrete regions of the viral genome, such as the ITAM motifs of the envelope transmembrane protein (TM), are particularly important for infection [22]. Contradicting the concept that retroviral genomes are highly condensed, sequences located between the env gene and the Tax/Rex boundary are dispensable for infection [23]. In particular, deletion of R3 and G4 preserves infectivity but affects replication efficiency [24]. Similarly, deletion of HTLV p12^I^ or p13^II^/p30^II^, the orthologs of BLV R3 and G4, impairs replication in macaques. In contrast, the mutations do not affect viral replication in rabbits, emphasizing the importance of relevant animal models [25]. R3 and G4 are nevertheless dispensable for pathogenesis, although their integrity contributes to disease frequency and latency [26].

Reverse genetics also generated unexpected observations, such as replication at wild-type levels of proviruses expressing fusion-deficient envelope proteins (TM A60V and A64S) [27]. These mutants are thus in principle unable to undergo an infectious cycle and may replicate preferentially through mitotic division of the host cell.

Mutations within the LTR further revealed that the viral promoter contained sub-optimal enhancers (AGACGTCA, TGACGGCA, TGACCTCA) that are essential for viral replication. As expected, site directed mutagenesis of the enhancers into consensus cyclic-AMP responsive elements (TGACGTCA) increases promoter efficiency but strongly impairs viral replication [28,29]. The presence of suboptimal enhancers in all BLV and HTLV-1 isolates suggests an evolutionary conserved mechanism that may reduce basal transcription and facilitate the escape from immune response.

Collectively, these observations thus emphasize the dichotomy between conclusions drawn from *in vitro* experimentations and their relevance in the animal model.

## 4. A Mutation that Increases Pathogenicity: Potential Hyperpathogenic Strain

Until recently, all mutations introduced in the BLV provirus were silent or at most reduced replication and pathogenesis *in vivo*. We recently reported that mutation of a N-linked glycosylation site (N230) affects the stability of the SU envelope protein and increases cell-to-cell transmission suggesting that this site restricts infectivity and viral replication [30]. A mutant carrying the N230 mutation replicates faster and is more pathogenic compared to the isogenic wild-type BLV strain. This observation thus suggests a mechanism of co-evolution restricting excessive pathogenicity that would indirectly impair mutual persistence of the virus and its host. Occurrence of this type of mutation may thus represent a potential threat associated with emergence of hyperpathogenic BLV strains and possibly also of new HTLV variants.

## 5. *In Vivo* Kinetics Indicates that BLV-Infected Cells Undergo High Turnover during Chronic Infection of Sheep 

The BLV model has been instrumental to understand the dynamics of cell turnover *in vivo*. In principle, lymphocyte homeostasis is the result of a critical balance between cell proliferation and death. Initial experiments using intravenous injection of bromodeoxyuridine (BrdU) demonstrated that B-lymphocytes are proliferating significantly faster in BLV-positive asymptomatic and persistently lymphocytotic sheep than in uninfected controls. In fact, an excess of 0.9% cells are produced by proliferation each day and, during leukemia, these rates even rise by up to tenfold [31]. Excess of cell proliferation was also reported in HTLV-induced HAM/TSP using a similar strategy based on incorporation of deuterated glucose [32]. In contrast, persistent lymphocytosis in BLV-infected cattle is characterized by a decreased B cell turnover resulting from a reduction of cell death and an overall impairment of proliferation, as observed in human chronic lymphocytic leukemia (CLL) [33,34].

Cell dynamics can also be estimated by intravenous injection of carboxyfluorescein diacetate succinimidyl ester (CFSE) [35]. Since CFSE labels proteins via their NH_2_ terminal ends, halving of fluorescence indicates that a cell has undergone cell division. Fitting cell numbers and fluorescence intensities revealed massive destruction of B-lymphocytes during chronic infection of sheep. In contrast, lymphocyte trafficking to and from lymphoid organs was unaffected.

Collectively, quantification of the dynamic parameters deduced from BrdU and CFSE kinetics shows that the excess of proliferation in lymphoid organs is compensated by increased death in peripheral blood [36]. Ablative surgery demonstrated that the spleen is a major lymphoid tissue massively destroying BLV-infected cells [37].

BLV chronic infection is thus characterized by a very dynamic equilibrium between a virus attempting to proliferate under a tight control exerted by the immune response.

## 6. Massive Depletion of Clones Located in Genomic Transcriptionally Active Sites during Infection

As retroviruses, BLV and HTLV-1 replicate via an infectious cycle upon expression of progeny virions as well as by mitotic division of provirus-carrying cells (clonal expansion). High throughput sequencing of proviral integration sites revealed the relative importance of these two cycles in viral replication varies during infection [38,39]. The majority of infected clones are created early before the onset of an efficient immune response. Two months from inoculation, the main replication route is mitotic expansion of pre-existing infected clones. Initially, BLV proviral integration significantly favors transcribed regions of the genome. Negative selection then eliminates 97% of the clones detected at seroconversion and disfavors BLV-infected cells carrying a provirus located close to a promoter or a gene. Nevertheless, among the surviving proviruses, clone abundance positively correlates with proximity of the provirus to a transcribed region. Two opposite forces thus operate during primary infection and dictate the fate of long term clonal composition: (1) initial integration inside genes or promoters and (2) host negative selection disfavoring proviruses located next to transcribed regions.

## 7. Tight Control of Virus-Positive Cells by the Immune Response

BLV infection is thus characterized by a massive depletion of provirus-carrying cell clones at early stages and a very dynamic turnover during chronic infection (Section 5). If the host immune response tightly controls viral replication, it is predicted that cells expressing viral antigens would be shorter lived. This question was addressed by comparing the survival rates of two cell pools isolated from the same donor and labeled with different fluorochromes depending on the absence or the presence of viral proteins induced *ex vivo* [40]. As predicted, transient viral expression significantly reduced the lifespan of BLV-infected lymphocytes. Cyclosporine treatment further supported the concept that an efficient immune response is required to control virus-expressing cells. This evidence is consistent with the presence of suboptimal LTR promoters that restrict viral reactivation (see Section 3) enabling escape from immune mediated destruction.

## 8. A Therapy Based on Activation of Viral Expression

BLV persistence is thus a very dynamic process characterized by a virus that continuously attempts to replicate and an active control exerts by the host immune response. As outlined in Section 2, viral proteins promote infectious and mitotic cycles but also expose the infected cell to immune control. Evidence for a very strong immune response is supported by the presence of virus-specific cytotoxic T cells and by high titers of neutralizing antibodies. Persistence of infected cells is thus possible providing that viral proteins are not expressed, perhaps under the control of viral microRNAs. In this context, we evaluated the therapeutic effectiveness of a strategy based on the induction of viral gene expression using valproic acid (VPA), a lysine deacetylase inhibitor [41,42]. VPA efficiently induced viral expression in primary cultures and reduced the number of leukemic cells in sheep. This strategy was then translated to another B cell neoplasm [43] and to HTLV-1 infected patients with HAM/TSP [44]. The treatment appeared to be safe but unable to permanently reduce proviral loads over the long term [45]. Instead, combination of VPA and other lysine deacetylase inhibitors with a standard regimen of ATL (AZT + IFN) is promising in ongoing clinical trials [46,47].

## 9. Towards an Efficient Vaccine

Except in the European Union, the herd prevalence of BLV worldwide ranges between 30% and 90% [48]. Major economic losses result from leukemia/lymphoma-induced death, reduction in milk production and custom restrictions. Thus, there is an urgent need for an efficient, safe and cost-effective vaccine against BLV. Previous vaccine candidates faced problems of efficacy (*i.e.*, only a fraction of animals were protected), persistence (*i.e.*, rapid decrease of immune protection), cost (e.g., production of purified proteins) or safety (e.g., genetically modified hybrid viruses). Therefore, we designed another approach based on a life-attenuated BLV strain harboring multiple deletions and mutations. The rationale was to delete pathogenic genes (*i.e.*, the oncogenic drivers) while maintaining a low level of infectivity. After a series of failures, we have identified a deleted BLV provirus that is infectious in cattle but replicates at very low levels. Inoculation of this vaccine elicits a vigorous anti-BLV immune response comparable to that of a wild-type infection. The vaccine does not spread to uninfected sentinels maintained during 7 years in the same herd and could not be detected in colostrum and milk from experimentally infected cows. Passive antibodies are transmitted to the newborn calves via the maternal colostrum. This anti-viral passive immunity persists during several months in the calves. However, the BLV mutant fails to transmit from cows to calves as assessed by nested PCR. In contrast to HIV, there is no significant sequence variation during infection [49,50]. Finally, vaccinated animals but not uninfected controls resist challenge by a wild type BLV virus. Trials are currently ongoing to evaluate the efficacy and safety of the vaccine in large herds in Argentina.

## 10. Conclusions

Understanding the mechanisms of BLV infection has provided valuable information on viral transmission, persistence and pathogenesis. In particular, reverse genetics yielded conclusions that could not be predicted from experiments performed *in vitro* as exemplified by a provirus containing an optimized promoter that was nevertheless attenuated. BLV persistence is characterized by a very dynamic cell turnover, which is rather unusual for a chronic infection. Host immunity is essential to control viral replication as indicated by surgical spleen ablation. Disruption of viral latency with epigenetic modulators has therapeutic value in BLV leukemia and may be useful for treatment of ATL. Finally, availability of an efficient anti-BLV vaccine is informative to develop preventive and curative measures in HTLV.
